# Treatment of end-stage lymphedema following radiotherapy for lymphoma: A case report

**DOI:** 10.1097/MD.0000000000025871

**Published:** 2021-05-14

**Authors:** Kyung-Chul Moon, In-Jae Yoon

**Affiliations:** Department of Plastic Surgery, Korea University College of Medicine, Seoul, South Korea.

**Keywords:** lymphedema, lymphoma, lymphovenous anastomosis, vascularized lymph node transfer

## Abstract

**Rationale:**

: Despite significant advances in microsurgical techniques, simultaneous vascularized lymph node transfer (VLNT) and lymphovenous anastomosis (LVA) surgeries may be effective for treatment of end-stage lymphedema. This case report describes the successful treatment of end-stage lymphedema with VLNT and LVA.

**Patient Concerns:**

A 72-year-old patient with bilateral lower extremity lymphedema was referred to our lymphedema clinic. This patient had a history of lymphoma and treated with radiotherapy on right inguinal area 26 years ago. Interestingly, the patient developed lymphedema on both the right and left lower extremities although she had radiotherapy on her right inguinal area.

**Diagnosis:**

According to the indocyanine green lymphography, lymphoscintigraphy, and magnetic resonance lymphangiography, the patient was diagnosed with end-stage lymphedema (International Society of Lymphology stage 3).

**Intervention:**

The patient underwent simultaneous VLNT and LVA for treatment of end-stage lymphedema.

**Outcomes:**

Significant reduction in circumference and volume of lower extremity was achieved following simultaneous VLNT and LVA

**Lessons:**

Simultaneous VLNT and LVA surgeries may be effective in patients with end-stage lymphedema.

## 1 Introduction

Lymphedema is a chronic edematous condition affecting approximately 1 in 30 people worldwide.^[1]^ The pathogenesis of lymphedema ensues predominantly from the modalities employed to treat cancer, particularly breast^[2,3]^ and gynecologic cancers,^[4]^ and radiotherapy.^[3]^ Lymphedema may increase medical, economic, and social burdens because lymphedema may persist patients despite combination treatment including decongestive therapy, diligent skincare, manual and mechanical lymphatic drainage, compression garments, laser therapy, and pneumatic compression devices.^[5]^ The longer lymphedema persists, the greater the possibility that serious complications such as cellulitis and sepsis will develop in patient, which can lead to hospitalization. Therefore, surgical treatment may be considered in patients who have exhausted conservative treatments.

Various surgical options are available to treat lymphedema, such as direct excisional debulking surgery, suction-assisted lipectomy debulking, lymphovenous anastomosis (LVA), and vascularized lymph node transplantation (VLNT).^[6]^ Direct excisional debulking surgery and suction-assisted lipectomy debulking are indicated for severe fibroadipose soft tissue hypertrophy. However, these traditional surgeries may result in unacceptable scarring and increase the risk of flap necrosis and recurrence. Recent advances in supermicrosurgery have evolved to treat lymphedema microsurgically. LVA is indicated for early-stage lymphedema and VLNT extends to indications for physiologic surgery in those with significant soft tissue excess resulting from chronic lymphedema.^[6]^

However, no studies have addressed simultaneous surgery with both LVA and VLNT for patients with advanced-stage lymphedema. This case report describes successful surgical treatment using simultaneous surgery of both LVA and VLNT for a patient with end-stage lymphedema.

## 2 Patients and methods

### 2.1 Patient

A 72-year-old female was referred to our lymphedema clinic with a 26-year history of chronic acquired left lower extremity lymphedema. She had a history of lymphoma and treated with radiation therapy on right inguinal area. Interestingly, she had lymphedema in both extremities, which was more severe in the left lower extremity (International Society of Lymphology stage 3) than in the right lower extremity (International Society of Lymphology stage 2). She first noticed the signs and symptoms of lymphedema after radiation therapy. She complained of severe left lower extremity heaviness and pain, difficulty in ambulation, and recurrent cellulitis, and intermittent sepsis that required hospitalization. She underwent combination treatment including decongestive physiotherapy for years, daily manual and mechanical lymphatic drainage, and compression garments. However, the nonsurgical management of lymphedema did not significantly improve the swelling and associated symptoms.

The patient did not have a history of diabetes mellitus or hypertension according to the complete patient medical history. The severity of the vascularity was measured by the ankle-brachial index, partial transcutaneous partial oxygen tension, and 3-dimensional computed tomography angiography. The patient had a decreased ankle-brachial index in both extremities and the transcutaneous partial oxygen tension on the left lower extremity was 8 mm Hg. The lower extremity computed tomography angiography identified severe stenosis of the bilateral superficial femoral artery. Furthermore, a left peroneal artery in the lower extremity was completely occluded below the knee. Percutaneous transluminal angiography was recommended but the patient strongly refused. Indocyanine green (ICG) fluorescence lymphography showed severe dermal backflow in the entire extremity including the foot and no lymphatic vessels were visualized on her left lower extremity with severe lymphedema. However, magnetic resonance lymphangiography showed some functioning lymphatic vessels were identified in the lower extremity with severe lymphedema. Therefore, we planned simultaneous LVA and VLNT for the left extremity that had severe lymphedema for 26 years and we provided detailed information on the donor sites except for the contralateral groin flaps including inguinal lymph nodes due to the high risk of aggravating the contralateral lower extremity lymphedema. The patient selected supraclavicular lymph node transfer and LVA and underwent surgery. Written informed consent was obtained from the patient.

### 2.2 Preoperative work-up

The ICG lymphography, lymphoscintigraphy, and magnetic resonance lymphangiography were performed before surgery. A milliliter of ICG mixed with 2% lidocaine was injected subcutaneously at the second web space of the affected extremity at the bedside. The fluorescence images of the lymphatic vessels were obtained with a near-infrared camera (Moment K; IANC&S, Seoul, South Korea), and the functioning lymphatic vessels were marked (Fig. 1 ). The functioning lymphatic vessels were not identified in the patient using the ICG lymphography and magnetic resonance lymphangiography was used to identify the functional lymphatic vessels for LVA. For supraclavicular VLNT, surface marking was made to form a triangle of the external jugular vein laterally, the lateral border of the sternocleidomastoid (SCM) muscle medially, and the clavicle inferiorly, containing supraclavicular lymph nodes to be harvested.

**Figure 1:**
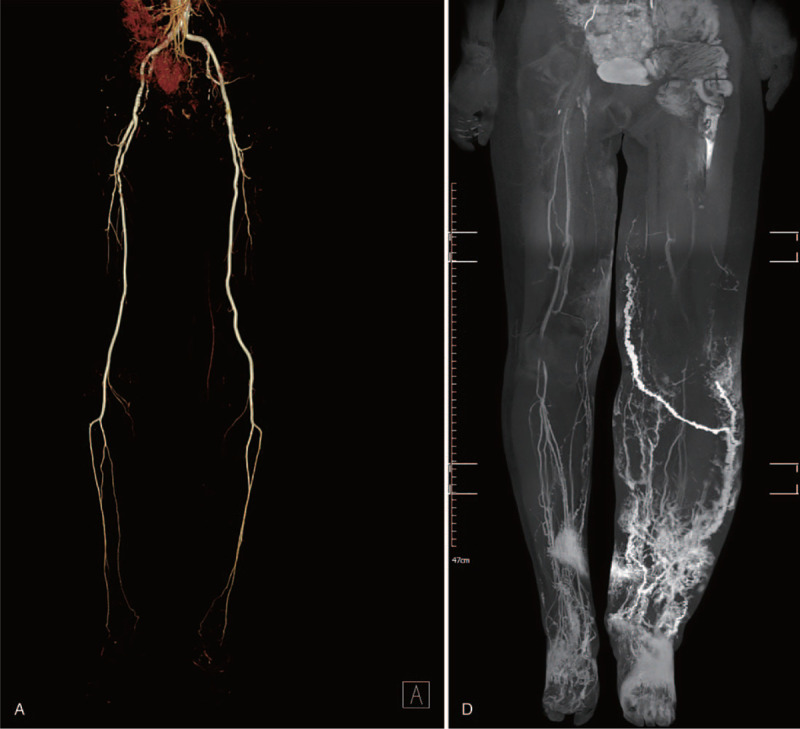
A patient with vasculopathy and severe lower extremity lymphedema. (A) Computed tomography angiography. (B) Indocyanine green lymphography. (C) Lymphoscintigraphy. (D) Magnetic resonance lymphangiography. This patient had International Society of Lymphology stage 3 lymphedema (end-stage lymphedema) with confluent dermal backflow and no functional lymphatic vessels were visualized in the indocyanine green lymphography.

**Figure 1 (Continued):**
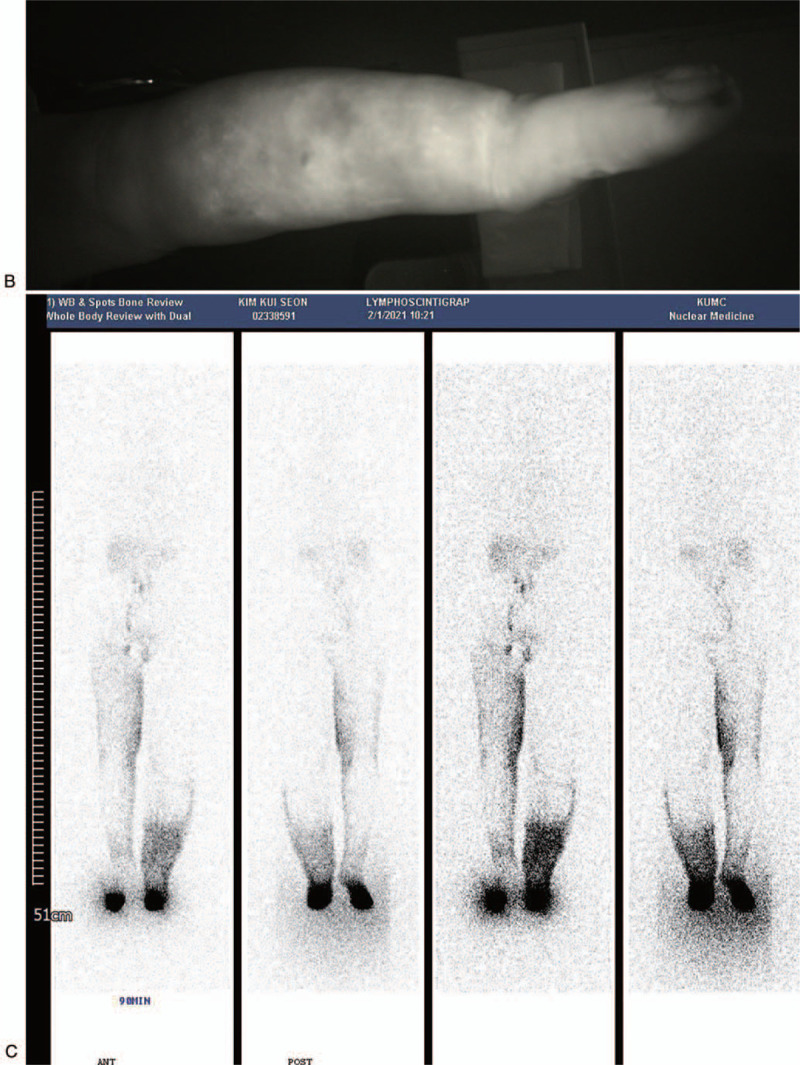
A patient with vasculopathy and severe lower extremity lymphedema. (A) Computed tomography angiography. (B) Indocyanine green lymphography. (C) Lymphoscintigraphy. (D) Magnetic resonance lymphangiography. This patient had International Society of Lymphology stage 3 lymphedema (end-stage lymphedema) with confluent dermal backflow and no functional lymphatic vessels were visualized in the indocyanine green lymphography.

The circumference of the affected and unaffected lower extremity was measured using a standardized measuring tape. The circumference of both the affected/unaffected extremities was measured in 6 places, 15 cm above the knee, 10 cm above the knee, at the knee (popliteal crease as the reference point as relevant), 10 cm below the knee, 15 cm below the knee, and the ankle. The circumference difference ratio was calculated according to the formula: (circumference of affected extremity − circumference of unaffected extremity)/circumference of unaffected extremity × 100. The volume of the extremity was also calculated based on the circumference measures. The volume segment was measured according to the formula of a truncated cone: *V* = *π* × *h* × (*R*^2^ + *r*^2^ + *Rr*)/3 where *π* is a constant, *h* is the height, *R* is the radius on base, and *r* is the radius on top.^[7,8]^ The circumferences of the extremity were measured 10 cm above the knee and 10 cm below the knee was used to calculate the volume segment. The volume of the unaffected extremity was also measured and the volume difference ratio was ultimately calculated according to the formula: (volume of affected extremity − volume of unaffected extremity)/volume of unaffected extremity × 100.^[9]^

### 2.3 Surgical technique

The VLNT surgery was performed first, followed by the LVA. Under general anesthesia, the patient was in the supine position with her head tilted 45° away from the right side of the harvest. A 5 cm skin incision was made 2 cm above the clavicle, and the omohyoid muscle near the SCM muscle was identified, dissected, and retracted using a rubber loop. Dissection of the supraclavicular flap proceeded carefully medially to laterally underneath the SCM muscle. The external and internal jugular vein and carotid artery were identified and meticulous dissection was performed to prevent massive bleeding. All submuscular fat including the lymph nodes were included in the flap with careful retraction of the external and internal jugular vein and carotid artery. The transverse cervical (TC) artery and vein were identified and dissected from the lateral to medially until the thyrocervical trunk was identified. After dissection of the TC pedicle, the supraclavicular flap was elevated from the anterior scalene muscle. The phrenic nerve, supraclavicular nerve, and right lymphatic duct were not injured. Once the flap elevation was done, the recipient vessels were identified using a Doppler and dissected. The TC pedicle was ligated, and the flap was transferred to the recipient site of the affected extremity. End-to-side anastomosis using 9-0 and 10-0 nylon sutures was performed for transplantation. Flap survival was confirmed by Doppler and flap bleeding in the visual field.

**Figure 2:**
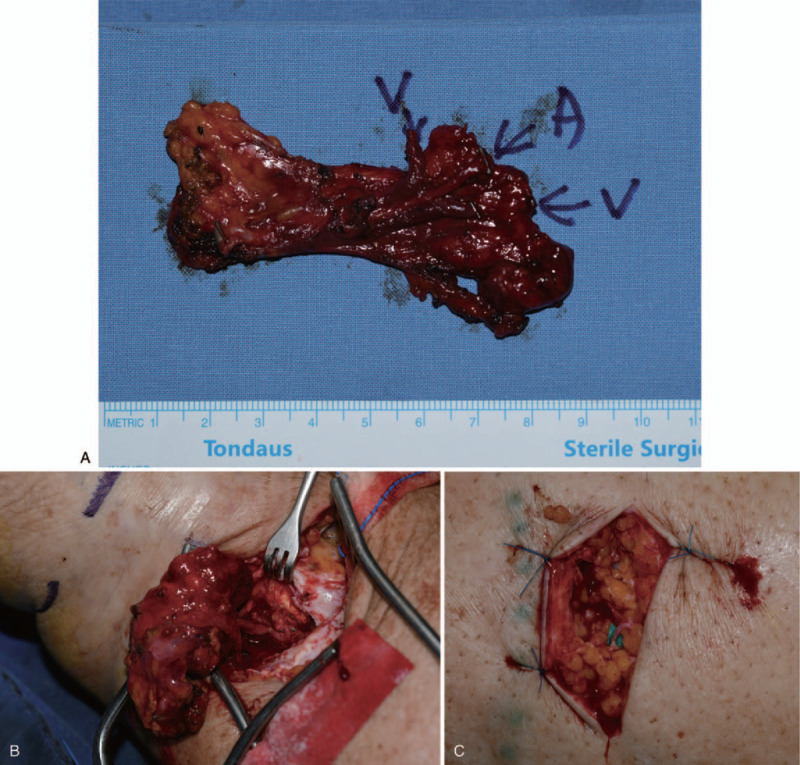
Simultaneous vascularized lymph node transfer and lymphovenous anastomosis surgeries. (A) After harvest of the supraclavicular flaps including 4 lymph nodes. (B) After end-to-side anastomosis at the ankle level. (C) After lymphovenous anastomosis. This patient underwent supraclavicular lymph node transfer, followed by lymphovenous anastomosis. Three lymphovenous anastomosis at the medial and lateral lower leg.

After the VLNT surgery, 2 to 3 lymphatic vessels were used for microsurgical anastomosis to the adjacent veins for LVA. Intraoperatively, 2 to three 3-cm longitudinal incisions were made under a surgical microscope according to the preoperative mapping based on the result of the magnetic resonance lymphangiography. After the superficial fascia incision, functioning lymphatic vessels were identified deep to the superficial fascia and 1 to 2 functional lymphatic vessels were anastomosed to the adjacent veins using 11-0 nylon sutures. Functional drainage was confirmed by washout of the venous blood in the anastomosed vein (Fig. 2).

### 2.4 Postoperative care

Antibiotics were administered to prevent surgical site infection. The affected extremity was compressed and elevated immediately postoperatively and postoperative compression bandage therapy with 35 to 40 mm Hg pressure was instituted for at least 6 months following surgery.

Three months after surgery, the patient had a significant volume reduction in the left lower extremity, which was obvious in her feet and lower leg. Her left lower extremity symptoms of heaviness and pain decreased. She had improved ambulation and required a lighter level of compression garments.

### 2.5 Clinical outcomes

**Figure 3:**
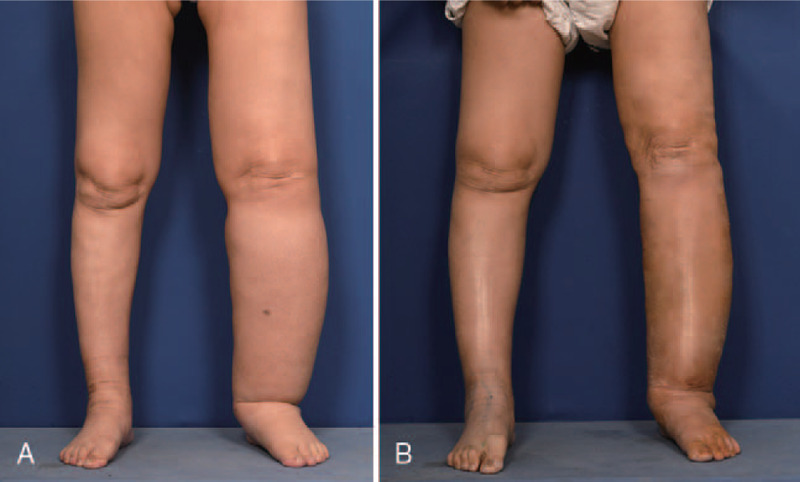
Preoperative and postoperative views of lower extremities (A) Preoperative view. (B) Three-month postoperative view. This resulted in significant reduction of the circumference and volume of the left lower extremity and the patient reported improvement in symptoms of lymphedema without complication.

The circumference difference ratios before surgery were 29%, 28%, 34%, 29%, 34%, and 32% at the levels of 15 cm above the knee, 10 cm above the knee, at the knee (popliteal crease), 10 cm below the knee, 15 cm below the knee, and at the ankle. The ratios were decreased to 25%, 23%, 18%, 15%, 14%, and 16% at the levels, respectively. The volume difference ratio was also decreased from 48% to 23%. The patient had significant the circumference and volume reduction of the left lower extremity (Fig. 3). Symptoms related to lymphedema improved following VLNT performed synchronously with LVA.

## 3 Discussion

The selection of the proper surgical method for lymphedema may be important to achieving favorable results. This study suggests that the extremity volume and circumference difference ratio could be reduced by simultaneous LVA and VLNT surgeries in a patient with the end-stage (stage 3) lymphedema. Traditionally, LVA may be considered for early-stage lymphedema (stage 1) and VLNT alone may show sufficient improvement in patients with the advanced stage lymphedema (stage 2 and 3). Where the entire extremity is severely affected by lymphedema, direct excisional debulking surgery, and suction-assisted lipectomy debulking surgeries have been recommended. Therefore, additional procedures such as lipectomy or liposuction are generally warranted in patients who underwent LVA or VLNT only. Simultaneous LVA and VLNT surgeries may be an alternative option to improve the clinical outcomes by enhancing the lymphatic drainage than VLNT alone.

Both LVA and VLNT are physiologic procedures to restore lymphatic fluid drainage of the affected area and have recently gained significant popularity for the surgical treatment of lymphedema. Previous observational studies supported the efficacy of LVA and VLNT for lymphedema in reducing lower extremity lymphedema volume and episodes of cellulitis.^[9–14]^ However, to the best of our knowledge, no studies have reported the surgical technique of simultaneous LVA and VLNT surgeries in patients with end-stage lymphedema.

The optimal flap for VLNT remains an area of considerable debate and we used supraclavicular flap as the donor site. The most serious complication of harvesting lymph nodes from the groin and axilla is the risk of devastating donor site lymphedema.^[15,16]^ In this study, the patient had lymphedema on the contralateral lower extremity, and thus, the contralateral groin flap could not be used for VLNT. Harvesting omental lymph nodes with the gastroepiploic pedicle may increase the risk of adhesion and subsequent bowel obstruction, while the submental flap risks damage to the marginal mandibular nerve and facial palsy, and has an unsightly scar.^[6,17]^ The supraclavicular VLNT including cervical level Vb lymph nodes may provide a ready supply of lymph nodes with minimal donor site morbidity.^[17,18]^ This is a thin and pliable flap suitable for distal extremity placement and the resultant scar can be well-concealed by clothing.^[6]^ The mean number of lymph nodes in supraclavicular flaps was reported to be 3.3 ± 1.5, comparable to the numbers in the groin and submental flaps.^[6]^ Where advanced-stage lymphedema predominantly affects the distal extremities as lymphatic fluid transport is severely impaired, heterotropic nonanatomical lymph node placement allows for the fluid to be absorbed from the most gravity-dependent position in the extremity.^[6]^ Therefore, we selected anterior tibial vessels of the ankle as the recipient-site for anastomosis.

In addition to VLNT, LVA may benefit patients with end-stage lower extremity lymphedema when functional lymphatic vessels can be identified.^[9]^ Recently, magnetic resonance lymphangiography and supermicrosurgery techniques become available to successfully identify functioning lymphatic vessels and demonstrated LVA results in improving lymphedema. Therefore, simultaneous VLNT and LVA surgeries following identifying functional lymphatic vessels and successful anastomosis with the adjacent veins may be a potential tool in the treatment of end-stage lymphedema.

This study showed that simultaneous VLNT and LVA surgeries could effectively reduce the circumference and volume of the lower extremity in a patient with end-stage lymphedema. Although a further investigation to compare long-term outcomes between simultaneous VLNT and LVA versus LVA or VLNT alone, the authors recommend simultaneous VLNT and LVA surgeries as the first treatment option for patients with end-stage lymphedema.

## Acknowledgments

The author is indebted to Eun-Sang Dhong, Deok-Woo Kim, and Eul-Sik Yoon who shared their experience in the harvest of the supraclavicular vascularized flap and microsurgical technique.

## Author contributions

**Conceptualization:** Kyung-Chul Moon.

**Data curation:** Kyung-Chul Moon, In-Jae Yoon.

**Formal analysis:** Kyung-Chul Moon.

**Funding acquisition:** Kyung-Chul Moon.

**Investigation:** Kyung-Chul Moon, In-Jae Yoon.

**Methodology:** Kyung-Chul Moon.

**Project administration:** Kyung-Chul Moon.

**Resources:** Kyung-Chul Moon, In-Jae Yoon.

**Software:** Kyung-Chul Moon.

**Supervision:** Kyung-Chul Moon.

**Validation:** Kyung-Chul Moon, In-Jae Yoon.

**Visualization:** Kyung-Chul Moon.

**Writing – original draft:** Kyung-Chul Moon, In-Jae Yoon.

**Writing – review & editing:** Kyung-Chul Moon, In-Jae Yoon.
